# *Panstrongylus guentheri* Berg, 1879 (Hemiptera, Reduviidae, Triatominae): first record in Mato Grosso, Brazil

**DOI:** 10.1590/0037-8682-0230-2024

**Published:** 2024-12-16

**Authors:** Mirian Francisca Martins, Sinara Cristina de Moraes, Sirlei Franck Thies, Veruska Nogueira de Brito, Rodrigo Gurgel-Gonçalves, Cleber Galvão

**Affiliations:** 1 Secretaria de Estado de Saúde de Mato Grosso, Vigilância em Saúde Ambiental, Barra do Garças, MT, Brasil.; 2 Secretaria de Estado de Saúde de Mato Grosso, Vigilância em Saúde Ambiental, Sinop, MT, Brasil.; 3 Secretaria de Estado de Saúde de Mato Grosso, Vigilância em Saúde Ambiental, Cuiabá, MT, Brasil.; 4 Universidade de Brasília, Laboratório de Parasitologia Médica e Biologia de Vetores, Campus Darcy Ribeiro - Asa Norte, Brasília, DF, Brasil.; 5 Instituto Oswaldo Cruz, Laboratório Nacional e Internacional de Referência em Taxonomia de Triatomíneos, Rio de Janeiro, RJ, Brasil.

**Keywords:** Chagas disease, New records, Cerrado, Surveillance programs, Trypanosoma cruzi

## Abstract

**Background:**

This paper reports the occurrence of *Panstrongylus guentheri* Berg, 1879 in the State of Mato Grosso (MT).

**Methods:**

Triatomines were captured in rural environments in municipalities of MT. Specimens were identified, and *Trypanosoma cruzi* infection was investigated.

**Results:**

Nineteen specimens of *Panstrongylus guentheri* were found, primarily inside houses. The geographical distribution of this species was recorded in nine municipalities of Mato Grosso, and parasitological tests did not detect *T. cruzi* on *P. guentheri*.

**Conclusions:**

The distribution of this species in Brazil has been recently updated.

Chagas disease, an infection caused by the protozoan parasite *Trypanosoma cruzi* (Chagas, 1909), has emerged as a risk to humans with the establishment of triatomine vectors in homes. Triatominae is a subfamily of Reduviidae (Hemiptera and Heteroptera), which includes bugs with morphological adaptations associated with host-seeking and blood-feeding^1^ ^,^ ^2^. These blood-sucking insects are vectors of *T. cruzi*, which is transmitted to humans and other mammals through feces and urine. The parasite can also spread through oral transmission, blood transfusions, organ transplants, or from mother to child. Therefore, even in the absence of triatomines in houses, the risk of transmission remains high. The subfamily Triatominae comprises 158 species, 155 extant and three extinct species, within 18 genera and five tribes^3^ ^,^ ^4^.

The genus *Panstrongylus* Berg, 1879, has a wide geographic distribution in the Neotropical region^2^. New reports of home invasions by *Panstrongylus* specimens have been documented in Brazil^5^. The highest diversity of *Panstrongylus* species was found in rainforest habitats^6^. They are found in rural, urban, and suburban areas with wildlife corridors and vector domiciliation. Species of *Panstrongylus* are potential vectors of *T. cruzi*. The increasing frequency of *Panstrongylus* species and the ability to invade and colonize human habitats is of interest to medical entomologists and Chagas disease control managers throughout Latin America^7^.

The genus was described based on *Panstrongylus guentheri* Berg, 1879, and comprises 16 extant species and one fossil species described^6^ ^,^ ^8^. In Brazil, 10 species of this genus have been recorded, with five occurring in the state of Mato Grosso^9^. These include *P. megistus* (Burmeister, 1835), *P. geniculatus* (Latreille, 1811), *P. rufotuberculatus* (Champion, 1899), *P. lignarius* (Walker, 1873)^10^ and *P. diasi* Pinto and Lent, 1946^11^. 

*Panstrongylus guentheri* has been documented in Argentina, Bolivia, Paraguay, and Uruguay. It occurs in the dry Chaco forests and savanna-grassland ecoregions of the Río de la Plata Basin^2^ ^,^ ^12^. In Brazil, it has been reported in the State of Mato Grosso do Sul^9^. This species has been observed in armadillo and rodent caves, as well as in association with opossums^1^. It has also been documented in the peridomicile, in proximity to woodpiles and environments frequented by goats and dogs^2^. 

From an epidemiological point of view, studies on the geographic distribution of these understudied vectors are crucial for understanding the epidemiological aspects of *T. cruzi* transmission and must be considered to guide disease control and monitoring. The present study describes the occurrence of *P. guentheri* in the state of Mato Grosso, Brazil. We present new records of *P. guentheri*, extend the distribution of species in the Cerrado areas, and present data on collection habitats and parasitological examinations.

In this study, the presence of *P. guentheri* was discovered for the first time in 2006 during the routine work of the Chagas Disease Control Program of Environmental Health Surveillance. One female and one male were collected from peridomiciles in the rural locality of Sítio Bom Jesus in the municipality of Várzea Grande, Mato Grosso, Brazil (15.541667N, 56.288333 W). 

The specimens of *P. guentheri* from Várzea Grande were identified by technicians at the Entomology Laboratory of the Secretaria de Estado de Saúde de Mato Grosso (SESMT) using the dichotomous keys of Lent and Wygodzinsky^1^. The specimens were deposited in the Entomology Laboratory of the SESMT and Triatominae Col­lection at the Oswaldo Cruz Institute (CTIOC) with registration number 12288. *Trypanosoma cruzi* infection was investigated using optical microscopy of live bugs in the laboratory. Therefore, this study included the geographical distribution of this species in the State of Mato Grosso, Brazil.

The identification of *P. guentheri* was based on the anterolateral processes of pronotum elongation and prominence. We also noted the upper surface of the head convex in the lateral view, the anterior and middle femora with more than three denticles, and the lateral margins of the pronotum lobes forming a distinct angle (Figure 1).

**FIGURE 1:**
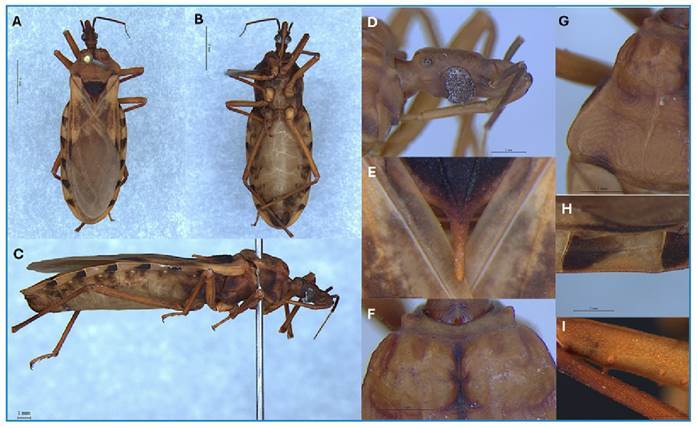
Male of *Panstrongylus guentheri* of Varzéa Grande, Mato Grosso State, Brazil. **A:** dorsal view. **B:** ventral view. **C:** lateral view, **D:** the orange-brown head, lateral view. **E:** apical process of the scutellum. **F:** tubercles discs in the anterior lobe of the pronotum. **G:** humeral angles rounded. **H:** intersegmental suture of the connexivum. **I:** denticles of the femora.

The female specimen of *P. guentheri* found in Várzea Grande had the following characteristics: length 27 mm; pronotum width 6 mm; and width of abdomen 9.5 mm. General color orange-brown, with dark brown areas on pronotum, scutellum, corion, and connexivum; apparently glabrous body surface. Head orange-brown, strongly raised between the eyes in dorsal view; one and a half times longer than the width between the eyes (1:0,6) and smaller than the pronotum (1:1,5); anteocular region twice as long as the postocular region (1:0.5); clypeus narrow, widened posteriorly; gena narrow, apically rounded; juga abruptly terminated; eyes in lateral view, distant from upper surface of head and extending below level of lower surface; ratio of width of eyes to distance between eyes 1:2.5. Ocellus surrounded by a dark grey ring. Antenna tubercle with small apicolateral process. The first rostral segment is close to the level of the anterior margin of the eyes; the second segment extends to the level of the base of the neck; the first and lower surfaces of the second rostral segment practically glabrous; upper surface of second and third segments with setae as long as or longer than diameter of segment; ratio of rostral segments 1:1.5:0.5. Pronotum pale brown with median groove, anterior and posterior margins dark brown; anterior lobe convex, irregularly rugose, disc tubercles rudimentary, lateral tubercles absent; posterior lobe transversely rough; submedian carinae low and fading in the posterior half; humeral angles rounded; anterolateral projection prominent and conical. The scutellum is dark brown and rough with shallow median depression; the posterior process is light brown, horizontal, cylindrical, apically rounded, and as long as the main body of the scutellum. Hemelytra extending to the seventh urotergite; clavus brown; corium dark brown except at the base, where it forms a light spot; membrane also light dark brown. Orange-brown legs: anterior and middle femora with more than two denticles visible, anterior femora with additional small denticles of irregular arrangement. 

Convex abdomen transversely striated, with discrete short setae and apparently glabrous median region; colored orange-brown, darkened on sides and genital region, dark reddish brown near connexivum tissue. Connexivum brown-reddish, anterior half or two-thirds of each connexival plate with a subrectangular dark brown spot adjacent to intersegmental suture; rudimentary spiracles from connexival suture; suture between rudimentary uroternites II and III.

Triatomine specimens were collected from the municipality of Várzea Grande, MT, during routine entomological surveillance between 2006 and 2023 (Figure 2). Since then, the presence of *P. guentheri* has been confirmed in other municipalities of the state. Nineteen specimens of *P. guentheri* were collected: 11 males, 5 females, and 3 unidentified. Microscopic examination of the feces of the five specimens did not reveal the presence of *T. cruzi* (Table 1). New records of *P. guentheri* have occurred, and currently, this species has been detected in nine municipalities in Mato Grosso in the Cerrado biome (Figure 2), between altitudes of 185 and 600 m (Table 1). 

**FIGURE 2:**
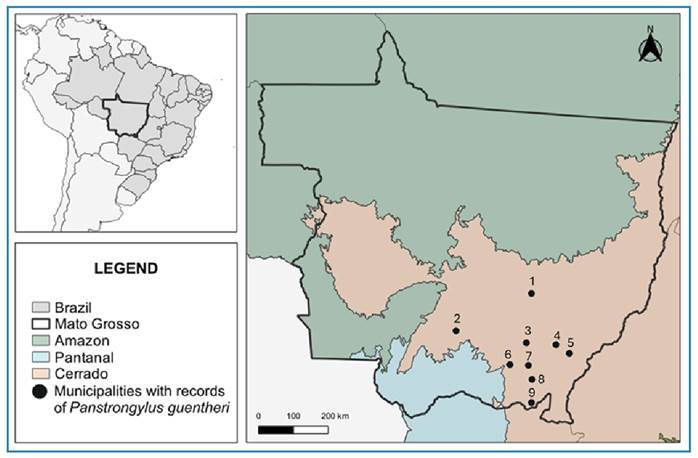
Municipalities of Mato Grosso, Brazil, with records of *Panstrongylus guentheri.* **1:** Paranatinga. **2:** Várzea Grande. **3:** Poxoréu. **4:** Tesouro. **5:** Guiratinga. **6:** Rondonópolis. **7:** São José do Povo. **8:** Pedra Preta **9:** Itiquira.

**TABLE 1:** Records of *Panstrongylus guentheri* in Mato Grosso, Brazil: geographical data, sex, habitats, and parasitological results.

Species	Sex	Year	Municipatily	Locality	Area	Environment	Parasitological diagnosis	Geographic coordinates	Altitude
*Panstrongylus guentheri*	Female	2006	Várzea Grande, MT	Sadia 3- Sítio Bom Jesus	Rural	Peridomicile	Unrealized	15.5416, 56.2883	185
*Panstrongylus guentheri*	Male	2006	Várzea Grande, MT	Sadia 3- Sítio Bom Jesus	Rural	Peridomicile	Unrealized	15.5416, 56.2883	185
*Panstrongylus guentheri*	NI	2009	Rondonópolis, MT	Res. Marechal Rondon	Urban	NI	NI	16.4713, 54.6371	212
*Panstrongylus guentheri*	NI	2010	Rondonópolis, MT	Gleba Cascata	Rural	NI	NI	16.4559, 54.3379	212
*Panstrongylus guentheri*	NI	2010	Guiratinga, MT	Novo Horizonte	Urban	NI	NI	16.3374, 53.7648	500
*Panstrongylus guentheri*	Male	2011	Rondonópolis, MT	Chácara Globo Recreio	Urban	Intradomicile	Negative	16.4966, 54.5810	212
*Panstrongylus guentheri*	Female	2012	São José do Povo, MT	Bandeirante	Urban	Intradomicile	Unrealized	16.466, 54.2521	281
*Panstrongylus guentheri*	Male	2013	Pedra Preta, MT	Centro	Urban	Intradomicile	Unrealized	16.6114, 54.4595	265
*Panstrongylus guentheri*	Male	2014	São José do Povo, MT	Sítio Pindaíba	Rural	Intradomicile	Negative	16.4614, 54.2549	212
*Panstrongylus guentheri*	Male	2015	Pedra Preta, MT	Morada do Condor	Rural	Intradomicile	Unrealized	16.6022, 54.4877	265
*Panstrongylus guentheri*	Male	2015	Tesouro, MT	Sítio São Judas Tadeu	Rural	Intradomicile	Unrealized	15.91940, 54.9107	390
*Panstrongylus guentheri*	Male	2016	Paranatinga, MT	Pacú Matrinxã	Rural	Peridomicile	Unrealized	14.4288, 54.0502	600
*Panstrongylus guentheri*	Male	2016	Rondonópolis, MT	Chácara Globo Recreio	Rural	Intradomicile	Negative	16.4344, 54.6153	212
*Panstrongylus guentheri*	Female	2017	Rondonópolis, MT	Jardim Brasília	Urban	Peridomicile	Negative	16.46533, 54.6205	212
*Panstrongylus guentheri*	Male	2019	Guiratinga, MT	Assentamento 2 Irmãos	Rural	Intradomicile	Unrealized	15.8893, 52.2606	500
*Panstrongylus guentheri*	Female	2021	Pedra Preta, MT	Fazenda Córrego da Onça	Rural	Intradomicile	Unrealized	16.6484, 54.6952	265
*Panstrongylus guentheri*	Male	2021	Paranatinga, MT	Garimpo novo	Rural	Intradomicile	Negative	14.6048, 54.0453	600
*Panstrongylus guentheri*	Female	2022	Itiquira, MT	Sítio Santa Bárbara	Rural	Extradomicile	Unrealized	17.2132, 54.1499	520
*Panstrongylus guentheri*	Male	2023	Poxoréu, MT	Vila Santa Terezinha	Urban	Intradomicile	Unrealized	15.8409, 54.3941	450

**NI:** Not Informed; **MT:** Mato Grosso. **Note:** All records of *P. guentheri* were within the Cerrado biome.

The geographical distribution of *P. guentheri* has expanded to the State of Mato Grosso, resulting in an updated distribution map of the species in Brazil, which now includes two states: Mato Grosso do Sul and Mato Grosso. Since the discovery of *P. guentheri* in Mato Grosso, there has been a notable expansion of its spatial distribution. This highlights the necessity for future studies to gain a deeper understanding of this occurrence and its epidemiological significance.

Notably, some of these new records broadened the altitudinal distribution of *P. guentheri* ^12^ ^,^ ^13^. Another important observation from these records was their strong association with the Cerrado biome. Lent and Wygodzinsky^1^ classified this species as wild type. However, it is important to note that specimens were recorded in both rural and urban areas.

Native triatomine species can invade and colonize both domestic and peridomestic environments. *P. guentheri* specimens were collected from and around houses. However, to date, only adult specimens have been observed in domiciles, corroborating the findings of Brito^14^ about house invasion by sylvatic triatomines^14^. Nevertheless, there was no evidence of intrahousehold colonization by this species, as the specimens were captured inside houses. In this study, it was observed that both male and female *P. guentheri* invaded houses. However, the number of males was slightly higher than that of females, which is in agreement with Brito et al.^14^. Natural infection of *P. guentheri* by *T. cruzi* was not observed in this study. However, other studies have reported *P. guentheri* is naturally infected with *T. cruzi* ^1^ ^,^ ^6^.

In conclusion, the geographic distribution of *P. guentheri* in the Cerrado area of Brazil was updated. It is recommended that a continuous surveillance program be implemented in the municipalities to monitor the occurrence of *P. guentheri* in the Mato Grosso and Mato Grosso do Sul States and in the surrounding areas of Brazil, Argentina, Bolivia, Paraguay, and Uruguay. Future studies that develop ecological niche models^15^ are necessary to analyze the potential geographical distribution of *P. guentheri* in these countries.
